# Baculovirus Vector-Based Varicella-Zoster Virus Vaccine as a Promising Alternative with Enhanced Safety and Therapeutic Functions

**DOI:** 10.3390/vaccines12030333

**Published:** 2024-03-20

**Authors:** Chanyeong Lee, Minjee Kim, Jungmin Chun, Sehyun Kim, Doyoung Yoon, Hyeondong Lee, Heewon Bang, Hee-Jung Lee, Hosun Park, Young Bong Kim

**Affiliations:** 1 Department of Biomedical Science and Engineering, Konkuk University, Seoul 05029, Republic of Korea; tcsrl97@konkuk.ac.kr (C.L.); mj0411@konkuk.ac.kr (M.K.); anananq@konkuk.ac.kr (J.C.); yoondy8345@konkuk.ac.kr (D.Y.); hdlee97@konkuk.ac.kr (H.L.); hwbang57@konkuk.ac.kr (H.B.); ziniga@konkuk.ac.kr (H.-J.L.); 2 KRBioTech, Seoul 05029, Republic of Korea; credible@konkuk.ac.kr; 3 Department of Microbiology, College of Medicine, Yeungnam University, Daegu 42415, Republic of Korea; hspark@ynu.ac.kr

**Keywords:** varicella-zoster virus, baculoviral vector vaccine, cell-mediated immunity, glycoprotein E, glycoprotein B

## Abstract

Varicella-zoster virus (VZV) poses lifelong risks, causing varicella and herpes zoster (HZ, shingles). Currently, varicella and HZ vaccines are predominantly live attenuated vaccines or adjuvanted subunit vaccines utilizing VZV glycoprotein E (gE). Here, we propose our vaccine candidates involving a comparative analysis between recombinant baculoviral vector vaccines (AcHERV) and a live attenuated vaccine strain, vOka. AcHERV vaccine candidates were categorized into groups encoding gE only, VZV glycoprotein B (gB) only, or both gE and gB (gE-gB) as AcHERV-gE, AcHERV-gB, and AcHERV-gE-gB, respectively. Humoral immune responses were evaluated by analyzing total IgG, IgG1, IgG2a, and neutralizing antibodies. Cell-mediated immunity (CMI) responses were evaluated by enzyme-linked immunospot (ELISPOT) assay and Th1/Th2/Th17 cytokine profiling. In the mouse model, AcHERV-gE-gB elicited similar or higher total IgG, IgG2a, and neutralizing antibody levels than vOka and showed robust VZV-specific CMI responses. From the perspective of antigens encoded in vaccines and their relationship with CMI response, both AcHERV-gB and AcHERV-gE-gB demonstrated results equal to or superior to AcHERV-gE, encoding only gE. Taken together, these results suggest that AcHERV-gE-gB can be a novel candidate for alleviating risks of live attenuated vaccine-induced latency and effectively preventing varicella during early stages of life while providing strong CMI for effective resistance against HZ and therapeutic potential in later stages of life.

## 1. Introduction

The varicella-zoster virus (VZV) (subfamily: *Alphaherpesvirinae*, genus: *Varicellovirus*, species: *Human alphaherpesvirus-3*), a lymphotropic and neurotropic virus with a highly specific human host, has a double-stranded DNA genome size of 125 kb and causes varicella and herpes zoster (HZ, shingles) [1]. Varicella, also known as chickenpox, primarily affects children, with a global annual incidence of at least 140 million cases. Severe complications necessitating hospitalization occur in about 4.2 million cases, leading to approximately 4200 varicella-related deaths annually [2]. Although varicella is primarily a childhood disease, VZV infection during this period establishes a latency in the dorsal root and cranial sensory ganglia. Following childhood, latent VZV can be reactivated to HZ. HZ is a painful rash resulting from the reactivation of VZV in the nerve ganglia. It can lead to complications such as postherpetic neuralgia (PHN), a persistent pain that significantly affects the quality of life [3,4]. This is known to be associated with a decrease in cellular immunity to VZV, particularly observed in cases with compromised immune systems, such as older individuals or those receiving immunosuppressive therapy, like transplant recipients or human immunodeficiency virus (HIV)-infected individuals [5,6]. In the global population aged 50 and above, the annual incidence rate of HZ is approximately 5–10 per 1000 individuals, and the incidence of HZ in lifetime when not vaccinated ranges from 20 to 30% [7,8,9].

Since the initiation of vaccination with the attenuated varicella vaccine strain vOka in 1974, Various vaccine candidates are currently taking the lead in the market, demonstrating significant efficacy in inducing immune responses against VZV. This is particularly evident in their ability to elicit robust defenses against VZV, solidifying their prominence in the field [10]. Given this situation, numerous vaccine candidates with noteworthy features and advantages have been developed and implemented to prevent varicella and HZ [11,12,13]. However, it is worthwhile to consider that, based on the characteristics of the VZV, the strategic approach to VZV should not be limited to prevention alone. Given the unpredictability of the latent presence of VZV and its potential to emerge at any time, a vaccine with therapeutic potential is required. Such a vaccine should not only be prophylactic but also a therapeutic immune response when latent VZV is reactivated, thereby guiding the immune system to effectively treat the virus.

Live attenuated vaccines and viral vector vaccines are well-established vaccine platforms, as both introduce foreign antigens into the body, leading to the induction of innate immune responses and subsequent activation of immune cells such as T cells and B cells. A common feature of these two platforms is their ability to induce robust immune responses. However, the live attenuated vaccine platform utilizes weakened viruses, mimicking natural infection processes, which can lead to sufficient acquisition of immunity but may raise safety concerns. In contrast, viral vector vaccines utilize viral vectors engineered to prevent replication or possessing such characteristics to deliver key antigen genes into the body. Consequently, viral vector vaccines can elicit specific and potent immune responses against the expressed key antigen proteins and are considered safe. However, the degree of immune response obtained may vary depending on the type of vector used.

The recombinant baculovirus-based DNA vector system (AcHERV) is a viral gene delivery system for a vaccine platform and demonstrates effective immune responses, not only humoral immunity but also cellular immunity against viruses such as severe acute respiratory syndrome coronavirus-2 and Middle East respiratory syndrome coronavirus in previous studies, with their efficacy assessed and validated as high-performance vaccines [14,15]. Because of numerous CpG motifs in their genome, this baculovirus-based vaccine system serves as an effective adjuvant that induces efficient immune responses without the need for additional additives [16,17,18,19]. In addition, the baculovirus vector does not encounter efficacy degrading issues related to unintended vector neutralization by preexisting antibodies in the host. Moreover, it is considered a safe vector for mammals because it does not replicate in mammalian cells, given its origin as a non-mammalian insect system [16,20].

In this study, we constructed recombinant baculovirus delivering VZV glycoprotein B (gB) and glycoprotein E (gE) genes to develop a next-generation VZV vaccine and compared it with the current live attenuated vaccine.

## 2. Materials and Methods

### 2.1. Cells

*Spodoptera frugiperda* 9 (Sf9, Invitrogen, Carlsbad, CA, USA) cells were cultured in Sf-900II medium (Invitrogen, USA) at 27 °C. Human embryonic kidney 293 TT cells (293TT; ATCC, Manassas, VA, USA) were cultured in Dulbecco’s modified Eagle’s medium (DMEM; Thermo Fisher Scientific, Waltham, MA, USA) at 37 °C. Human lung fibroblast MRC-5 cells (ECACC, Salisbury, UK) were cultured in minimal essential medium (MEM; Gibco, Grand Island, NY, USA) at 37 °C.

### 2.2. Virus Preparation and Titration

VARIVAX (DQ008355) vaccine strain (vOka) in the cell-associated state was propagated in MRC-5 cells by infecting MRC-5 cells with a multiplicity of infection (MOI) of 0.005 and harvested 3 days post infection (d.p.i). For the cell-free state of vOka, MRC-5 cells were infected by cell-associated state vOka with MOI of 0.2 and harvested 2 d.p.i. with changing culture media to storage buffer (storage buffer; 5% sucrose, 10% FBS in PBS). Harvested cells were sonicated with 15 s on/15 s off cycle 5 times at 50% amplitude and clarified by centrifugation 3000× *g* for 30 min at 4 °C. Titration of cell-associated or cell-free state of vOka was conducted by plaque assay, serially diluted virus was inoculated MRC-5 cells and stained with crystal violet at 5 d.p.i. MRC-5 and vOka were kindly provided by Dr. Ho Sun Park (Yeungnam University, Daegu, Republic of Korea). Obtaining a cell-free state of vOka was conducted as previously described with some modifications [21]. All experiments were performed at the Biosafety Level 2 facility of Konkuk University (Republic of Korea; KUIBC-2023-05).

### 2.3. Construction of Recombinant Baculoviruses: AcHERVs

**Figure 1:**
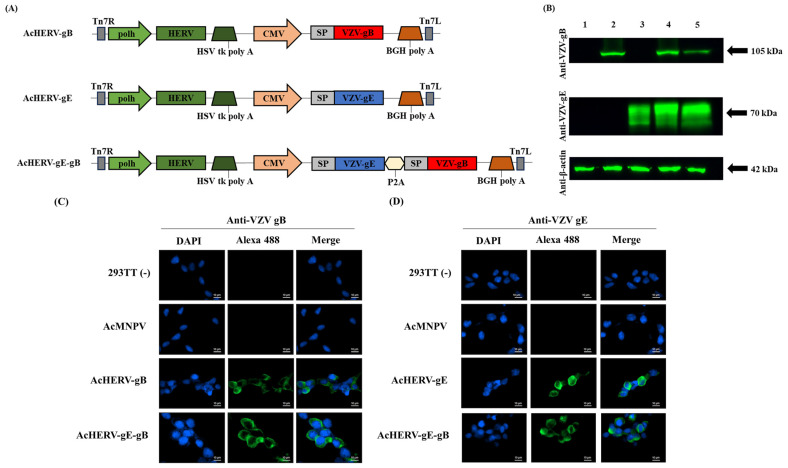
Characterization of VZV vaccine candidate using recombinant baculovirus-based vector. (**A**) Schematic diagrams of VZV vaccine candidates. (**B**) Expression of VZV-gB and VZV-gE in 293TT cells analyzed by Western blotting, normalized to β-actin expression. Lane 1: uninfected 293TT cells, Lane 2: AcHERV-gB, Lane 3: AcHERV-gE, Lane 4: AcHERV-gE-gB, Lane 5: inactivated VZV lysate. Expression and localization of (**C**) VZV-gB and (**D**) VZV-gE in 293TT cells infected with recombinant baculoviruses analyzed by immunofluorescence assay, detected by Alexa 488. Scale bar for all images is 10 µm. Abbreviation in Figure 1a: polh: polyhedrin promoter; HERV: human endogenous retrovirus envelope protein; HSV tk poly A: herpes simplex virus thymidine kinase poly A; CMV: cytomegalovirus promoter; BGH poly A: bovine growth hormone poly A; Tn7L and Tn7R: left and right end of transposition site; SP: signal peptide sequence; P2A: linker sequence originated by porcine teschovirus-1.

A recombinant baculoviral vector expressing HERV env (pFastBac1-HERV) was constructed by incorporating a codon-optimized synthetic gene of HERV type W envelope (GenBank accession number NM014590; GenScript, Piscataway, NJ, USA) into pFastBac1 (Invitrogen, USA). VZV-gE and VZV-gB (YC01, Korea strain, GenBank accession number KJ767491) were synthesized, respectively, with codon-optimizing procedure for optimum expression in mammalian cells (GeneArt, San Francisco, CA, USA) and then individually cloned into the pFastBac1-HERV vector under the CMV promoter, resulting in pFB-HERV-gE, pFB-HERV-gB, and pFB-HERV-gE-gB. To verify whether the gene was cloned at the accurate location without any sequence alterations, capillary electrophoresis sequencing was performed (Macrogen, Seoul, Republic of Korea). The pFastBac1 without any foreign gene was used for wild-type baculovirus (AcMNPV). For producing recombinant baculoviruses and AcMNPV, the Bac-to-Bac baculovirus expression system was utilized following the manufacturer’s instructions (Invitrogen, USA). A strategy for constructing recombinant baculoviruses AcHERV-gB, AcHERV-gE, and AcHERV-gE-gB is shown in Figure 1a. These three recombinant baculoviruses (AcHERV-gB, AcHERV-gE, and AcHERV-gE-gB; refer to AcHERVs) and AcMNPV were then propagated in Sf9 cells. AcHERVs and AcMNPV were purified by removing debris and virus-infected cells by centrifugation at 6000× *g* for 20 min at 4 °C. Supernatants were cushioned with 30% sucrose solution and centrifuged at 16,000× *g* at 4 °C for 1.5 h in an R15A rotor with HIMAC high-speed refrigerated centrifuge (HITACHI, Tokyo, Japan). Titration of baculovirus was carried out by quantitative polymerase chain reaction (qPCR) using the BacPAK qPCR Titration Kit (Takara Bio USA Inc., San Jose, CA, USA) following the guidelines provided by the manufacturer. Confirmation of VZV glycoprotein gene insertion into recombinant baculoviruses was carried out by M13 PCR and insert-specific PCR.

### 2.4. Western Blot and Immunofluorescence Assay

The expression of the VZV-gE and VZV-gB proteins in mammalian cells was tested by infecting 293TT cells with an MOI of 30. At 3 d.p.i., cells were harvested, and the expressed protein in cells was extracted utilizing PRO-PREP protein extraction solution (iNtRON Biotechnology, Seongnam, Republic of Korea). Each harvested protein was separated by size using SDS-PAGE gel electrophoresis and transferred to the membrane. Non-specific binding of antibody was blocked with 5% skim milk in tris-buffered saline, and monoclonal antibodies against VZV-gE (1:5000, Abcam, Cambridge, UK) and VZV-gB (1:1000, Biorbyt, Cambridge, UK) were bound for 1 h at room temperature (RT). After the secondary antibody binding step, detection and analysis of the target protein were conducted by ChemiDoc imaging systems (Bio-Rad, Hercules, CA, USA). Uncropped figures are available in the Supplementary Figure S3. Expression and localization of the VZV-gE and VZV-gB proteins were tested by infecting 293TT cells and Sf9 cells, respectively. At 2 d.p.i., cells were fixed with 4% formaldehyde solution, followed by permeabilization and blocking. Using the same antibodies as those used in the Western blot as the primary antibody, expressed protein in cells was detected by Alexa Fluor 488-conjugated secondary antibody. Nuclear staining was performed using DAPI. Analysis of the target protein was carried out using a BioTek Lionheart FX automated microscope (Agilent, Santa Clara, CA, USA).

### 2.5. Immunization of BALB/C Mice

The six-week-old female BALB/c mice (Orient-Bio, Seongnam, Republic of Korea) were bred in a controlled environment with the highest filtration standards, and they were provided unrestricted access to both water and food. All animal experiments were carried out in adherence to the National Institute of Health’s Guide for the Care and Use of Laboratory Animals and approved by the Institutional Animal Care and Use Committee of the Konkuk University (IACUC approval no. KU-23071). Mice were immunized by intramuscular injection into the hind legs with AcHERV-gB, AcHERV-gE, AcHERV-gE-gB, and AcMNPV (vehicle control) in PBS and by subcutaneous injection into the skin of the back with the cell-free state of vOka. Six mice per group were immunized twice at three-week intervals according to the doses specified in Table 1.

### 2.6. Enzyme-Linked Immunosorbent Assay

Blood samples were collected two weeks after the second immunization through the jugular vein following anesthetizing the mice by intramuscular injection Zoletil50 (40 mg/kg, Virbac Laboratories, Carros, France) and Rompun (5 mg/kg, Bayer Korea, Seoul, Republic of Korea). The induction of antibodies specific to VZV was tested using ELISA. To detect the VZV-specific antibodies, a 96-well plate was coated with cell-free VZV. The plate was then blocked with 5% skimmed milk in PBS for 1 h at 37 °C and treated with 1/200 diluted mouse serum for 2 h. The plate was washed with 0.05% tween-20 (Sigma-Aldrich, St. Louis, MO, USA) in PBS (PBS-T), treated with HRP-conjugated goat anti-mouse IgG (1:10,000; Abcam, UK), IgG1 (1:1000; Invitrogen, Carlsbad, CA, USA), IgG2a (1:400; Invitrogen, Carlsbad, CA USA) (anti-mouse IgG1 and IgG2a antibody were used same concentration for coating) antibody for 1 h at 37 °C, washed, and treated with TMB solution (Bio-Rad Laboratories, Hercules, CA, USA). An Epoch microplate reader (BioTek Instruments, Winooski, VT, USA) was used to measure the absorbance at 450 nm. Each experiment was repeated twice.

### 2.7. Neutralization Assay

FAMA assay was conducted according to William’s method with some modifications [22]. Briefly, MRC-5 cells were infected with VZV at an MOI of 0.005. Upon reaching 70–80% cytopathic effect (CPE), infected cells were harvested and washed with PBS. Serially diluted (2-fold) serum samples were prepared and incubated with infected cells for 30 min at room temperature (RT). The cells were washed and treated with Alexa Fluor 488 goat anti-mouse IgG (1:1000; Invitrogen, Carlsbad, CA, USA) for 30 min at RT. After washing, the cells were mounted on slides, treated with a mounting solution containing DAPI, covered with a coverslip, and observed under a fluorescence microscope (NIS-Elements-BR, Nikon, Tokyo, Japan). Titer was calculated as the reciprocal of the highest dilution factor, showing a bright fluorescent ring on the cell surface, with a titer of 4 or higher considered positive.

### 2.8. Enzyme-Linked Immunospot Assay

**Figure 2:**
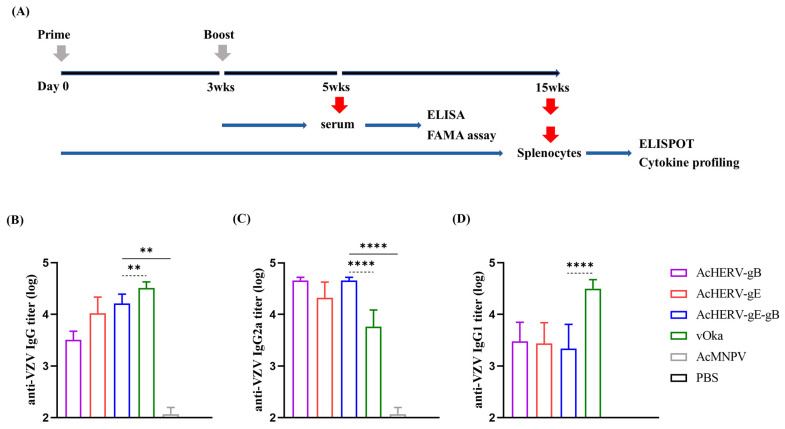
Humoral immune response of AcHERVs immunization in BALB/c mice. (**A**) Vaccination and immunogenicity assessment schedule. (**B**) Anti-VZV-specific total IgG antibody responses in immunized groups, (**C**) anti-VZV-specific IgG2a antibody responses in immunized groups, and (**D**) anti-VZV-specific IgG1 antibody responses in immunized groups; assessments are determined by ELISA. *p*-values were generated using one-way ANOVA followed by Dunnett’s multiple comparisons tests, ****: *p* < 0.0001, **: *p* < 0.01, compared with the AcMNPV group (solid lines) and the vOka group (dotted lines). The error bars represent the standard deviation (SD).

Twelve weeks after the second immunization, the spleen was isolated after sacrificing mice through carbon dioxide gas (Figure 2a). The isolated intact spleen was washed clearly with PBS and pressed into a cell strainer. For collecting splenocytes, strained cells were incubated with Red Blood Cell Lysing Buffer (Sigma-Aldrich, USA) and washed with PBS. Detection of interferon-gamma (IFN-γ) secreted from splenocytes of immunized mice was carried out by enzyme-linked immunospot assay (ELISPOT; BD Bioscience, Franklin Lakes, NJ, USA), as described by the manufacturer’s instruction. Briefly, a 96-well plate for ELISPOT was coated with anti-mouse IFN-γ (0.5 μg/mL) and blocked with complete RPMI-1640 medium at 37 °C. Splenocytes were plated at a density of 1 × 10^6^ cells per well and stimulated by exposure to inactivated cell-free VZV (250 PFU/well) for 24 h at 37 °C. The plates were rinsed with PBS-T and treated with 0.25 μg of biotinylated anti-mouse IFN-γ detection antibodies for 2 h at RT. After a subsequent washing step, streptavidin-HRP was introduced, and color development was carried out using an AEC substrate reagent from BD Biosciences, USA. Spot quantification was performed using an ELISpot reader (AID Ispot EliSpot FluoroSpot Reader, AID Autoimmun Diagnostika GmbH, Straßberg, Germany), and the number of VZV-specific IFN-γ-secreting cells was calculated by subtracting the number of spots in negative control (stimulation with mock-infected MRC-5 lysate) from the number of spots in stimulation with VZV.

### 2.9. Cytokine Profiling

Cytokine profile analysis in immunized mice was conducted using cytometric bead array kits specific for mouse Th1/Th2/Th17 cytokine (BD bioscience, USA) following the manufacturer’s instructions. Splenocytes (1 × 10^6^ cells/well) were stimulated with inactivated cell-free VZV (250 PFU), and mock-infected MRC-5 lysates were used as a negative control. After incubation for 1 day, the culture supernatants of splenocytes were collected for cytokine detection. The concentration of VZV-specific cytokines was calculated by subtracting the concentration of the negative control (stimulation with mock-infected MRC-5 lysate) from the concentration of stimulation with VZV.

### 2.10. Statistical Analysis

Statistical analyses were conducted utilizing GraphPad Prism 8.0.2 (GraphPad Software Inc., San Diego, CA, USA), and data are expressed as means ± standard deviation (SD). Group comparisons were carried out through one-way analysis of variance (ANOVA), followed by Dunnett’s multiple comparison tests. Statistical significance was established at *p* < 0.05.

## 3. Results

### 3.1. Expression and Localization of VZV Antigens Delivered via Baculoviral Vector: AcHERVs

AcHERV-based VZV vaccine candidates were constructed, delivering the VZV glycoprotein genes, gE and gB (AcHERV-gE, AcHERV-gB, and AcHERV-gE-gB), and expressing under CMV promoter in mammals. To ensure that the protein expressed from the delivered gene is located in a suitable position to induce an immune response, human CD5 signal peptide sequences were added upstream of VZV glycoprotein genes (Figure 1a). Gene insertion into the baculovirus genome was confirmed by M13 PCR analysis and insert-specific PCR of viral DNA (Supplementary Figure S1).

Transduced gene expression of VZV-gE and VZV-gB are shown in Figure 1b. Each VZV antigen was observed at approximately 70 kDa for VZV-gE and 105 kDa for VZV-gB. Despite a considerably short exposure time, a satisfactory level of expression was observed in all candidates. In AcHERV-gE-gB, dual genes were expressed at similar levels to those of the single gene-delivering baculoviruses, AcHERV-gE, or AcHERV-gB.

Through the immunofluorescence assay, we confirmed the intracellular localization of expressed VZV glycoprotein via recombinant baculovirus vector. In mammalian cells, specifically 293TT cells, expression of the VZV-gB and VZV-gE proteins were detected throughout the cytoplasm, with slightly more accumulation observed in regions closer to the cell membrane (Figure 1c,d). In insect cells, we confirmed that VZV glycoprotein was not expressed under the CMV promoter (Supplementary Figure S2).

### 3.2. Humoral Immune Response by AcHERVs

VZV-specific IgG levels were evaluated by ELISA. The mean IgG titers for each immunized group were as follows: AcHERV-gB: 3200 (±1371.1), AcHERV-gE: 10,500 (±10,044.4), AcHERV-gE-gB: 16,200 (±7636.8), and vOka: 32,400 (±9353.1). Among immunized groups, total IgG titers of the AcHERV-gE-gB dual antigen group showed a synergistic effect by the addition of VZV-gB but less than the vOka group (Figure 2b). However, this result was presumed to be due to the use of cell-free vOka as the ELISA coating antigen for total IgG analysis.

To assess the contribution of immune responses to the Th1 response, IgG2a levels were evaluated. In contrast to the data for total IgG, a notably higher level was observed in the AcHERVs immunization groups than in the vOka group, with the AcHERV-gE-gB group showing the highest value. The mean IgG2a titers for each immunized group were as follows: AcHERV-gB: 45,900 (±6037.4), AcHERV-gE: 21,000 (±19,763.6), AcHERV-gE-gB: 45,900 (±6037.4), and vOka: 5816.6 (±5807.6) (Figure 2c).

Conversely, regarding IgG1, the vOka group showed a higher level than the AcHERVs immunization groups. The mean IgG1 titers for each immunized group were as follows: AcHERV-gB: 3000 (±3662.4), AcHERV-gE: 2733.3 (±3795.9), AcHERV-gE-gB: 2166.6 (±3877.1), and vOka: 31,500 (±14,428.1) (Figure 2d).

In conclusion, it was found that the group immunized with AcHERV-gE-gB constructed in this study induced higher Th1 immunity than vOka.

### 3.3. Neutralizing Capacity Evaluation of AcHERVs

**Figure 3:**
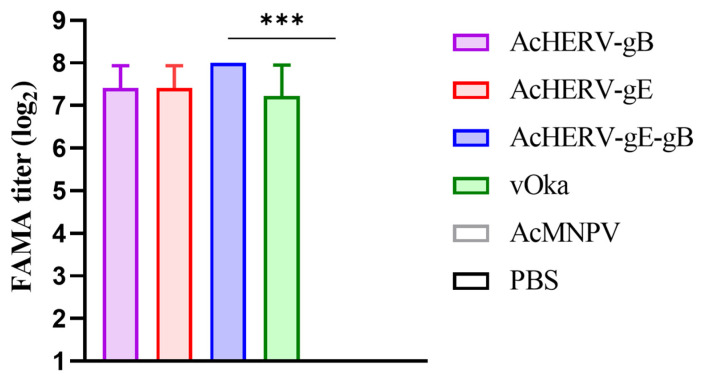
Neutralizing capability of AcHERVs immunization in BALB/c mice. VZV-specific neutralizing antibody titers in immunized groups performed by FAMA assay. Titer was calculated as the reciprocal of the highest dilution factor, showing a bright fluorescent ring on the cell surface, with a titer of 4 or higher considered positive. *p*-values were generated using one-way ANOVA followed by Dunnett’s multiple comparisons tests, ***: *p* < 0.001, compared with the AcMNPV group. The error bars represent the SD.

The level of VZV-specific neutralizing antibodies was evaluated using the FAMA assay, which is known for its sensitivity in capturing the distinctive features of VZV. The FAMA assay is employed as a reference method in the development of a new assay for VZV, and it is regarded as the gold standard, supplanting the plaque reduction neutralization test [23,24]. The mean FAMA titers for each immunized group were as follows: AcHERV-gB: 170.7 (±60.3), AcHERV-gE: 170.7 (±60.3), AcHERV-gE-gB: 256 (±0), vOka: 149.3 (±79.8), and AcMNPV: 2 (±0). A significant increase in FAMA titers was observed in the AcHERVs and vOka groups compared to the AcMNPV group, especially the AcHERV-gE-gB group, which showed the highest neutralization titers (Figure 3).

### 3.4. Cell-Mediated Immunity Response by AcHERVs

**Figure 4:**
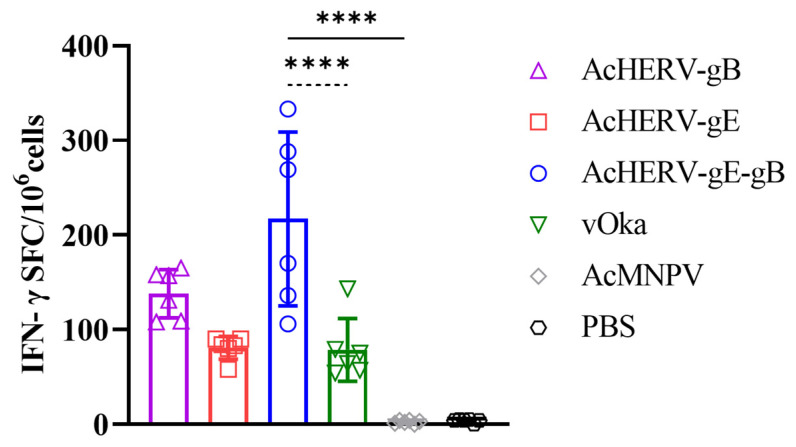
VZV-specific CMI response of AcHERVs immunization in BALB/c mice. VZV-specific CMI response secreting IFN-γ as shown by spot-forming cells (SFCs). The assay was performed 12 weeks after the second immunization by ELISPOT using splenocytes of immunized groups. *p*-values were generated using one-way ANOVA followed by Dunnett’s multiple comparisons tests, ****: *p* < 0.0001, compared with the AcMNPV group (solid lines) and the vOka group (dotted lines). The error bars represent the SD.

ELISPOT analysis was performed to evaluate the cell-mediated immunity (CMI) response of the AcHERVs and vOka immunized groups. Among the immunized groups, the AcHERV-gE-gB group exhibited the highest level of IFN-γ responses. The mean spot-forming cells (SFCs) per 10^6^ cells for each immunized group were as follows: AcHERV-gB: 138 (±23.4), AcHERV-gE: 80.8 (±10.8), AcHERV-gE-gB: 217 (±84), vOka: 78.7 (±30.1), and AcMNPV: 2.3 (±1.5) (Figure 4).

To confirm the broad spectrum of cellular immune cytokines induced by vaccine candidates, a cytometric bead array (CBA) was carried out. Among the Th1 cytokines, AcHERV-gE-gB exhibited the highest values among the immunized groups and coincided with those of the ELISPOT data. Regarding IL-2, the mean concentration values of each immunized group were as follows: AcHERV-gB: 70.3 (±20.7), AcHERV-gE: 68.7 (±21.9), AcHERV-gE-gB: 87.1 (±32.7), vOka: 83 (±47.9), and AcMNPV: 10.5 (±14.9). Concerning IFN-γ, the mean concentration values were as follows: AcHERV-gB: 495.3 (±263.2), AcHERV-gE: 159.2 (±68.7), AcHERV-gE-gB: 1011.8 (±246.8), vOka: 636.4 (±405.2), and AcMNPV: 20.2 (±13.3). Additionally, for TNF-α, the mean concentration values were as follows: AcHERV-gB: 106 (±30.2), AcHERV-gE: 94 (±35.5), AcHERV-gE-gB: 219.2 (±57.6), vOka: 122.5 (±11.4), and AcMNPV: 33 (±46).

The AcHERV-gE-gB group did not show a significant difference among Th2 cytokines, unlike Th1. However, the overall values were similar to those of the vOka group. The mean cytokine concentration values across each immunized group were as follows: For IL-4, AcHERV-gB: 44.3 (±19.3), AcHERV-gE: 45 (±17.8), AcHERV-gE-gB: 47.2 (±20.6), vOka: 31.2 (±14.9), and AcMNPV: 8 (±11.1). For IL-6, AcHERV-gB: 96 (±10.2), AcHERV-gE: 80.4 (±22.5), AcHERV-gE-gB: 109.2 (±14.9), vOka: 113 (±54.1), and AcMNPV: 30 (±42.2).

Lastly, for the Th17 cytokine, the mean IL-17A concentration values across each immunized group were as follows: AcHERV-gB: 45 (±24.4), AcHERV-gE: 45.2 (±8.5), AcHERV-gE-gB: 73.1 (±6.6), vOka: 37 (±5.5), and AcMNPV: 5.2 (±7.4).

**Figure 5:**
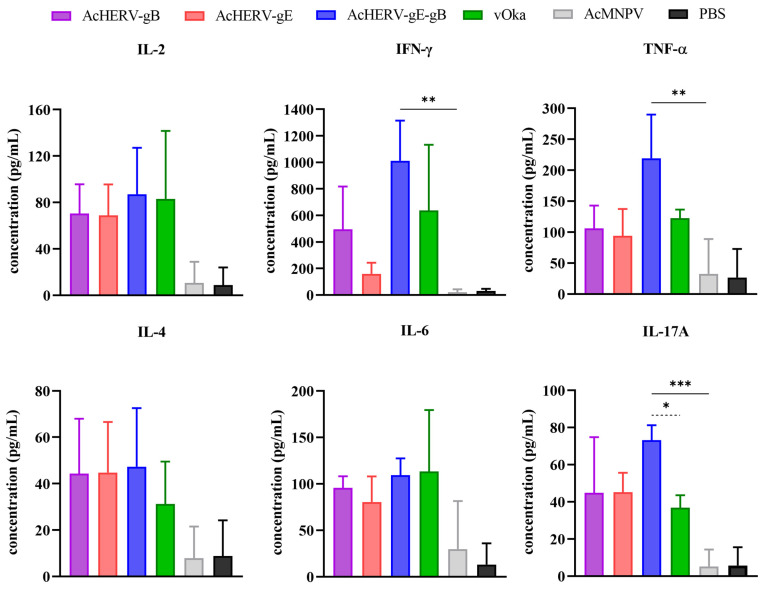
Cytokine profiles in VZV-specific CMI response of AcHERVs immunization in BALB/c mice. VZV-specific CMI responses were measured by cytokine secretion after stimulating splenocytes with VZV. The measurement of secretion levels was performed using a cytometric bead array for concentration detection. Upper row graphs represent Th1 cytokines (IL-2, IFN-γ, TNF-α), and lower row graphs represent Th2 (IL-4, IL-6) and Th17 (IL-17A). Each graph represents the secretion levels of specified cytokine in each immunized group. *p*-values were generated using one-way ANOVA followed by Dunnett’s multiple comparisons tests, ***: *p* < 0.001, **: *p* < 0.01, *: *p* < 0.05, compared with the AcMNPV group (solid lines) and the vOka group (dotted lines). The error bars represent the SD.

Among the immunized groups, the AcHERVs immunized groups showed comparatively higher Th1 response than those of the vOka group (Figure 5). Taken together, AcHERV-gE-gB induced not only humoral but also CMI responses against VZV.

## 4. Discussion

From the perspective of virus lethality, VZV does not possess a high fatality rate compared to other infectious viruses. However, it poses a sustained threat throughout an individual’s life [7,8]. Once a latent infection is established, it tends to be reactivated in immunocompromised states, such as aging-related immune weakening or immunosuppressive treatments in transplant recipients [5,6,25]. Shingles is a viral disease that is caused by the reactivation of a latent virus rather than an external viral infection, so regardless of specific preventive measures or social systems, many people infected with chickenpox in childhood suffer reactivation symptoms, and various complications occur.

Currently, antiviral agents for the treatment of infections caused by herpesvirus family members, including VZV, are acyclovir, valacyclovir (pro-drug form of acyclovir), and famciclovir. Antiviral medications should be promptly administered to suppress viral replication and reduce the duration and severity of HZ symptoms [3]. However, these treatments are temporary solutions for viral infections; thus, fundamental prevention is required. In the realm of vaccination against varicella, live attenuated zoster vaccine and recombinant VZV-gE subunit vaccine with liposome AS01B adjuvant are leading the global market [26,27,28,29,30].

In this study, a comparative analysis was conducted between the AcHERV system-based baculovirus vector VZV vaccine and the live attenuated VZV vaccine, which currently dominates the market, represented by the vOka vaccine strain. This comparative analysis revealed that the VZV vaccine developed through the AcHERV system elicits a robust immune response, demonstrating equivalent or superior efficacy compared to vOka. From a safety perspective, the system overcomes potential drawbacks associated with live attenuated VZV vaccines by non-replicating features in mammals. The comparative analysis with the protein vaccine, a prominent vaccine platform alongside live attenuated vaccines, is not included in this study. Protein vaccines usually require adjuvants as they face challenges in eliciting a strong immune response on their own [31]. Hence, in the context of developing protein vaccines, further research is warranted to explore the development of adjuvants capable of eliciting potent immune responses. Moreover, the vaccine’s effectiveness should be conducted in further studies to provide a comprehensive understanding of its performance. The results of such comparisons would require careful examination to draw definitive conclusions.

In terms of the developmental focus of vaccines in these circumstances, our research aimed to develop a new vaccine platform that is safe and has not just prophylactic efficacy but also strong CMI for fundamental therapeutic efficacy and alleviating the successive global burden of disease. Among the immunized groups, the AcHERVs immunized groups showed comparatively higher Th1 response than those of the vOka group. When comparing the details between the AcHERVs groups, the group immunized with AcHERV-gE, which encodes VZV-gE (abundant glycoprotein of VZV and a key focus of many studies [11,12,13]), exhibited relatively lower values than AcHERV-gE-gB. At this point, this study focused on VZV-gB, which has received less research attention than VZV-gE as an antigen for VZV vaccine development. AcHERV-gE-gB and AcHERV-gB, which encodes VZV-gB, demonstrated immune responses that were not inferior to those of AcHERV-gE and showed higher values, particularly in experiments associated with Th1-related responses. These observations suggest that a detailed investigation into the cellular immunogenicity of VZV-gB for protection against VZV is necessary. Notably, the established role of VZV-gB in syncytium formation via cell-to-cell fusion and viral egress implies that a vaccine incorporating VZV-gB, which triggers effective cellular immune responses, can impede viral clearance during infection or its reactivation by limiting viral spread within the host [32,33,34]. In brief, this prompts the need for closer examination, especially considering VZV-gB and its synergistic effect with VZV-gE, for vaccine development from the perspective of effective strategies concerning the VZV life cycle involving infection and reactivation [35].

It may be challenging to determine that a vaccine biased toward either humoral or cellular immunity alone can effectively lead to defense against VZV. To prevent varicella infection, robust humoral and cellular immunity are essential. For the protection against HZ, cellular immune responses, as well as humoral immune responses or, furthermore, a combination of these responses, is needed, like Natural Killer Cell Antibody-Dependent Cellular Cytotoxicity (NK-ADCC), especially considering the potential of HZ reactivation throughout our lifetime [36,37]. Therefore, there is a need for vaccine development that aligns more with the categories of prevention and treatment rather than prevention alone. Therefore, future VZV vaccines should focus on inducing a robust cellular immune response, leveraging the host’s immune system to prevent viral entry, and ensuring safety without vaccine-induced infections.

Unlike other viruses, the human-specific nature of VZV makes it challenging to apply methods, such as direct challenge experiments, in small animal models to assess protection against the virus [38,39]. Therefore, our research team indirectly examined the humoral and cellular immune responses. In future studies, developing small animal models with specific mechanisms for VZV entry will enable us to conduct challenge experiments using VZV, allowing the direct evaluation of vaccine efficacy. To mimic a state resembling VZV infection in a small animal model, tumor cells manipulated to overexpress VZV glycoprotein were transplanted into mice. Subsequent evaluation of the degree of tumor regression served as a means of assessing the therapeutic efficacy of the vaccine. Therefore, the development of a new animal model incorporating these examples would enable a more direct and clear evaluation of VZV vaccine development.

When considering potential issues with viral vector systems based on recombinant baculovirus, recombination associated with foreign genes is commonly cited as a concern. Additionally, challenges related to stability and capability arise alongside infectivity loss during passaging and scale-up processes [40,41]. Our research team was also aware of these risks and took steps to address them in this study. We were able to overcome these issues by securing the seed virus through plaque purification and conducting passaging tests on the recombinant baculovirus-based vaccine used in this study. Therefore, along with the preceding discussions, our research team proposes AcHERV-gE-gB as a promising candidate for the study and practical application of a vaccine against VZV.

## 5. Conclusions

In conclusion, through the balanced induction of both humoral and cellular immune responses in groups immunized with AcHERV-gE-gB, we present a novel vaccine candidate that holds promise for comprehensive immunity against varicella and effective viral clearance against HZ reactivation in a prophylactic or therapeutic manner. Furthermore, by leveraging the non-replicating nature of the AcHERV system in mammalian organisms, this recombinant baculovirus vector-based vaccine demonstrated potential as an efficient and safe alternative in the global VZV vaccine development landscape, reducing the global burden of VZV.

## Figures and Tables

**Table 1:** Animal experiment design for evaluating immune response against VZV.

Groups	Construction		Immunization		No. of Mice
		Dose	Interval	Route of Administration	
Group 1	AcHERV-gB	4 × 10^7^ FFU	3 weeks, 2 times	Intramuscular	6
Group 2	AcHERV-gE	4 × 10^7^ FFU	3 weeks, 2 times	Intramuscular	6
Group 3	AcHERV-gE-gB	4 × 10^7^ FFU	3 weeks, 2 times	Intramuscular	6
Group 4	vOka	2000 PFU	3 weeks, 2 times	Subcutaneous	6
Group 5	AcMNPV	4 × 10^7^ FFU	3 weeks, 2 times	Intramuscular	6
Group 6	PBS	-	3 weeks, 2 times	Intramuscular	6

## Data Availability

All data used during the study are available from the corresponding author upon request.

## Supplementary Materials

The following supporting information can be downloaded at: https://www.mdpi.com/article/10.3390/vaccines12030333/s1, Figure S1: Identification of target gene insertion into recombinant baculovirus genome. (A) PCR amplification of recombinant baculovirus viral DNA using M13 primer (B) PCR amplification of recombinant baculovirus viral DNA using insert specific primer. PCR conditions as follows were used: for M13 PCR, 95 °C for 3 min followed by 35 cycles at 95 °C for 30 s, 55 °C for 30 s, 72 °C for 8 min, and final extension at 72 °C for 1 min and for inset specific PCR, 95 °C for 3 min followed by 35 cycles at 95 °C for 30 s, 55 °C for 30 s, 72 °C for 3 min (VZV-gB) or 2 min (VZV-gE), and final extension at 72 °C for 1 min. M: DNA ladder, Sample No. 1: PCR negative control, No.2: wild-type baculovirus, No.3: AcHERV-gB, No.4: AcHERV-gE, No.5: AcHERV-gE-gB. Figure S2: Immunofluorescence assay detecting expression of VZV-gB and VZV-gE in Sf9 cells. Expression and localization of (A) VZV-gB and (B) VZV-gE in Sf9 cells infected with recombinant baculoviruses analyzed by immunofluorescence assay, detected by Alexa 488. Scale bar for all images is 10 μm. Figure S3: Whole image of western blot. Samples used in the western blot for each target protein were applied equally to each lane. Same membrane was cut and used for detection of each protein separately using anti-VZV-gE and anti-β-actin. M: protein marker, Lane 1: uninfected 293TT cells, Lane 2: AcHERV-gB, Lane 3: AcHERV-gE, Lane 4: AcHERV-gE-gB, Lane 5: inactivated VZV lysate.
