# Supplementary material for: Baculovirus Vector-Based Varicella-Zoster Virus Vaccine as a Promising Alternative with Enhanced Safety and Therapeutic Functions
Source: Vaccines (Basel). 2024 Mar 20;12(3):333. doi: 10.3390/vaccines12030333 (PMC10975603; doi:10.3390/vaccines12030333)
Supplement: Supplementary file 1 [file vaccines-12-00333-s001.zip › vaccines-2897507-supplementary.pdf]

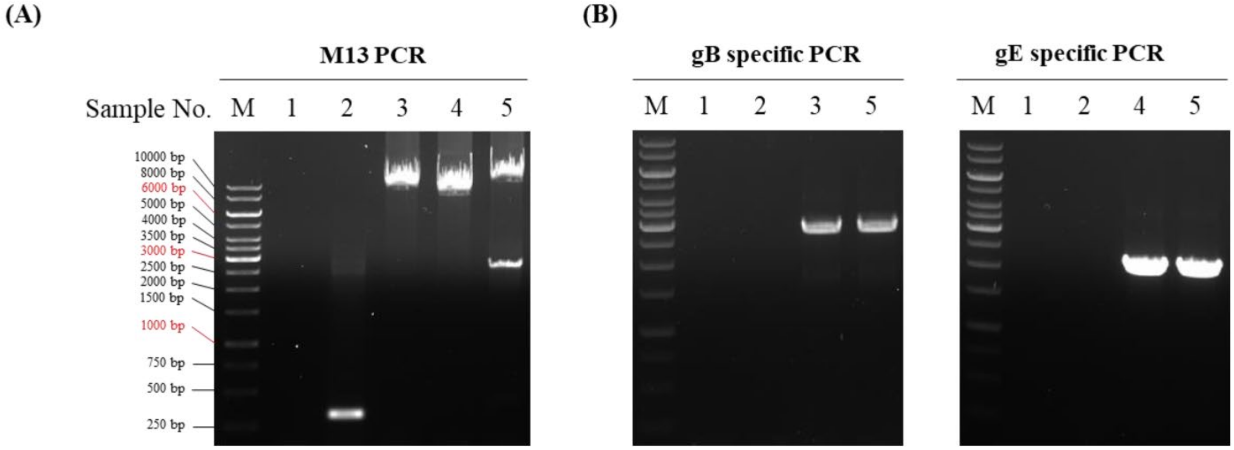

Supplementary Figure S1. Identification of target gene insertion into recombinant baculovirus genome. (A) PCR amplification of recombinant baculovirus viral DNA using M13 primer (B) PCR amplification of recombinant baculovirus viral DNA using insert specific primer. PCR conditions as follows were used: for M13 PCR, 95˚C for 3 min followed by 35 cycles at 95˚C for 30 sec, 55˚C for 30 sec, 72˚C for 8 min, and final extension at 72˚C for 1 min and for inset specific PCR, 95˚C for 3 min followed by 35 cycles at 95˚C for 30 sec, 55˚C for 30 sec, 72˚C for 3 min (VZV-gB) or 2min (VZV-gE), and final extension at 72˚C for 1 min. M: DNA ladder, Sample No. 1: PCR negative control, No.2: wild-type baculovirus, No.3: AcHERV-gB, No.4: AcHERV-gE, No.5: AcHERV-gE-gB.

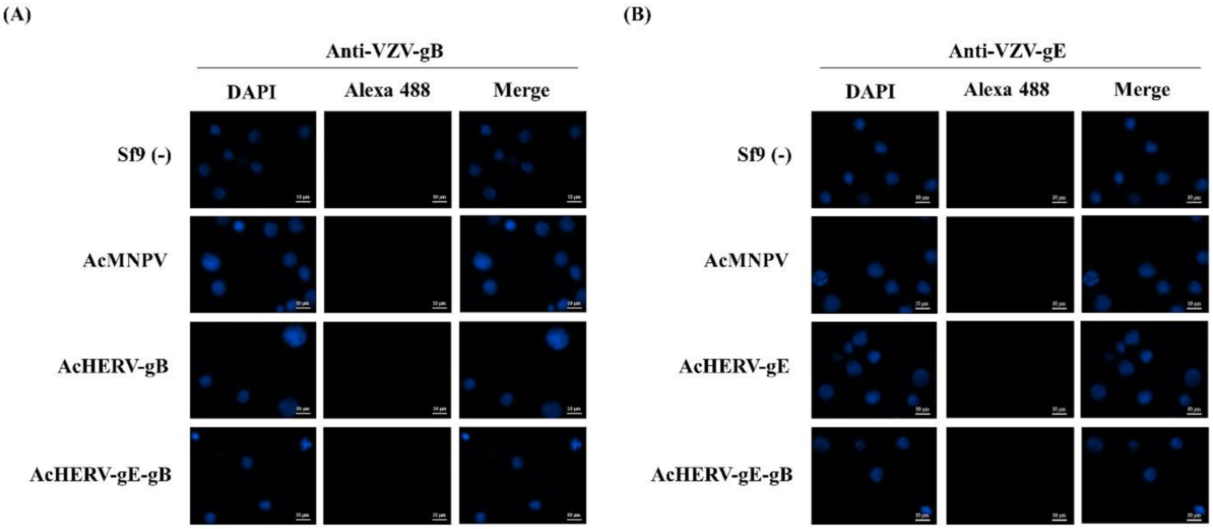

Supplementary Figure S2. Immunofluorescence assay detecting expression of VZV-gB and VZV-gE in Sf9 cells. Expression and localization of (A) VZV-gB and (B) VZV-gE in Sf9 cells infected with recombinant baculoviruses analyzed by immunofluorescence assay, detected by Alexa 488. Scale bar for all images is 10 μm.

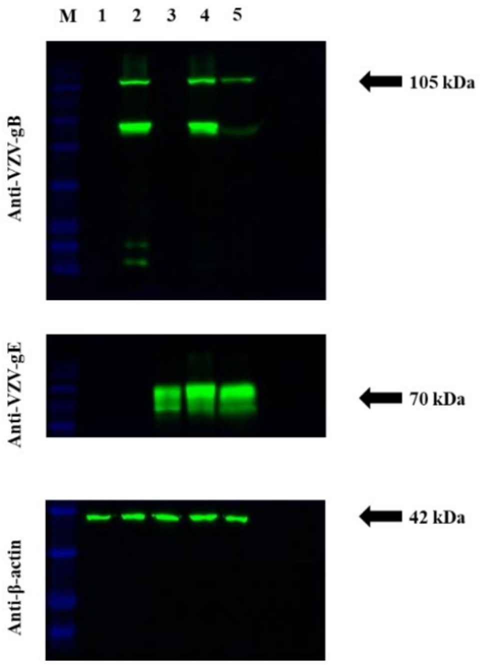

Supplementary Figure S3. Whole image of western blot. Samples used in the western blot for each target protein were applied equally to each lane. Same membrane was cut and used for detection of each protein separately using anti-VZV-gE and anti-β-actin. M: protein marker, Lane 1: uninfected 293TT cells, Lane 2: AcHERV-gB, Lane 3: AcHERV-gE, Lane 4: AcHERV-gE-gB, Lane 5: inactivated VZV lysate.
